# The future of clinical trials in idiopathic pulmonary fibrosis

**DOI:** 10.1097/MCP.0000000000001099

**Published:** 2024-07-04

**Authors:** Paolo Spagnolo, Toby M. Maher

**Affiliations:** a Respiratory Disease Unit, Department of Cardiac, Thoracic, Vascular Sciences and Public Health, University of Padova, Padova, Italy; b Department of Pulmonary, Critical Care and Sleep Medicine, University of Southern California Keck School of Medicine, Los Angeles, California, USA; c Section of Inflammation, Repair and Development, Imperial College London National Heart and Lung Institute, London, UK

**Keywords:** clinical trials, endpoints, Idiopathic pulmonary fibrosis, investigational drugs, treatment

## Abstract

**Purpose of review:**

Idiopathic pulmonary fibrosis (IPF) is a progressive lung disease with a poor prognosis and limited therapeutic options. A multitude of promising compounds are currently being investigated; however, the design and conductance of late-phase clinical trials in IPF has proven particularly challenging.

**Recent findings:**

Despite promising phase 2 data, ziritaxestat, an autotaxin inhibitor, pentraxin-2, an endogenous protein that regulates wound healing and fibrosis, and pamrevlumab, a human monoclonal antibody against connective tissue growth factor, failed to show efficacy in phase 3 trials. Endpoint selection is critical for the design, execution, and success of clinical trials; recently, attention has been paid to the assessment of how patients feel, function, and survive with the aim of aligning scientific objectives and patient needs in IPF. External control arms are control patients that derive from historical randomized controlled trials, registries, or electronic health records. They are increasingly used to assess treatment efficacy in clinical trials owing to their potential to reduce study duration and cost and increase generalizability of findings.

**Summary:**

Advances in study design, end point selection and statistical analysis, and innovative strategies for more efficient enrolment of study participants have the potential to increase the likelihood of success of late-phase clinical trials in IPF.

## INTRODUCTION

Idiopathic pulmonary fibrosis (IPF) is a relentlessly progressive and irreversible fibrotic lung disease of unknown cause associated with disabling symptoms, poor survival, and substantial healthcare utilization [1,2]. Recent data from the UK suggests an increased burden in the incidence of IPF with inevitable impact on health service planning and resource allocation [3]. Following two decades of negative clinical trials, pirfenidone and nintedanib, two drugs with pleiotropic antifibrotic effects, were approved worldwide based on their ability to slow disease progression, although neither drug halts nor reverses lung fibrosis [4,5]. In addition, neither drug improves symptoms or quality of life, and both are associated with significant tolerability issues. The IPF community is therefore in desperate need of more efficacious and better tolerated therapies.

In recent years, several high-quality clinical trials have been conducted in IPF. These studies have provided important insights into trial design and choice of endpoints. Despite high hopes of success, however, three promising potential IPF therapies, ziritaxestat, pentraxin-2 and pamrevlumab, all recently failed, leading to considerable disappointment and frustration [6]. Here, we summarize the most recent phase III trials in IPF and discuss how advances in study design, end point selection and statistical analysis may provide valuable insight into drug development and increase the chance of success of late-phase clinical trials in this devastating disease. 

**Box 1:**
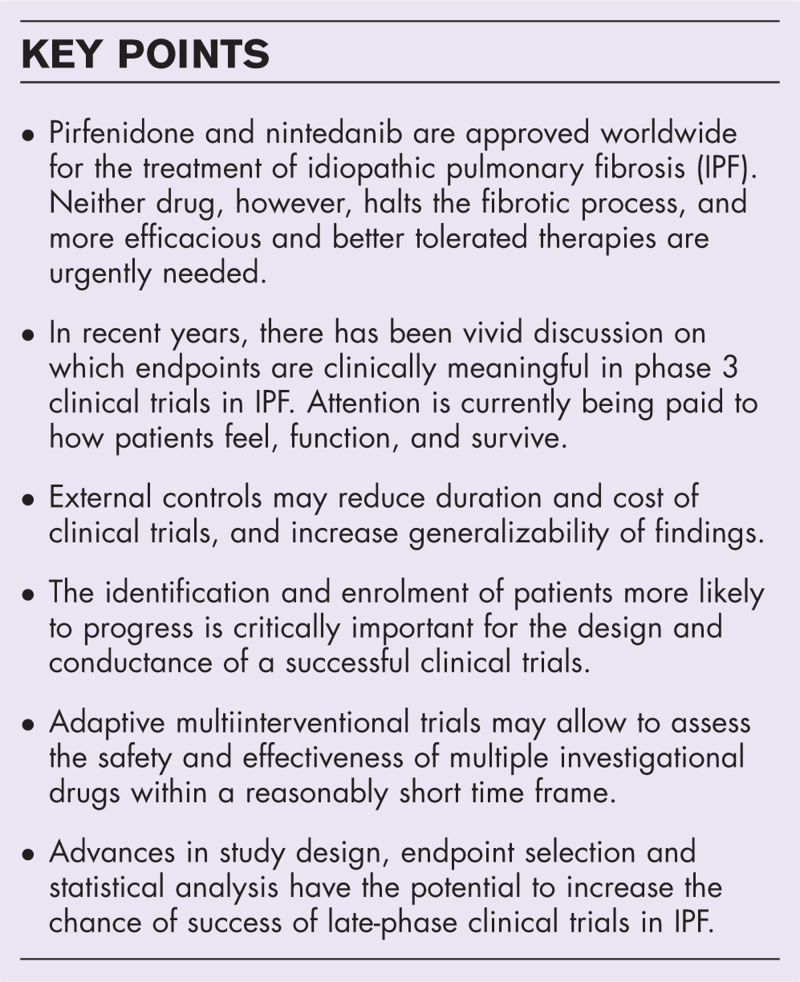
no caption available

## RECENT (NEGATIVE) PHASE III RANDOMIZED CONTROLLED TRIALS

*ISABELA 1 and ISABELA 2*. Ziritaxestat is a small molecule that selectively inhibits autotaxin, an enzyme involved in the production of lysophosphatidic acid, a profibrotic mediator that is upregulated in patients with IPF [7,8]. Following a 23-patient phase IIa study demonstrating effective target engagement and a smaller decline in forced vital capacity (FVC) vs. placebo at week 12 [9], the efficacy and safety of ziritaxestat were further evaluated in two identical phase III trials, ISABELA 1 and ISABELA 2 [10]. Main inclusion criteria included an FVC ≥45% and a diffusing capacity of the lung for carbon monoxide (DL_CO_) ≥30% of the predicted values. In both studies, patients were randomized 1 : 1 : 1 to receive ziritaxestat 600 mg, ziritaxestat 200 mg, or placebo once daily in addition to pirfenidone or nintedanib (or neither treatment). The primary outcome was the rate of decline in FVC at week 52. The two trials were terminated early following a planned review of pooled unblinded data indicating that ziritaxestat did not reduce the rate of decline of FVC and was associated with increased all-cause mortality rates compared to placebo. Similarly, ziritaxestat failed to show benefit in several secondary efficacy outcomes. Notably, in patients receiving standard of care, lung function did not decline at a slower rate than in untreated patients, and the annual rate of decline in FVC was greatest in patients taking pirfenidone, although ziritaxestat did not affect pirfenidone concentration. Furthermore, dose reductions and interruptions of nintedanib were more common in patients taking high-dose ziritaxestat, and dose reductions or interruptions of the study drug were more common in those taking nintedanib, with these findings being more pronounced in the ISABELA 2 trial, which enrolled a higher proportion of Asian patients. At the time of trial termination, 525 patients had been randomized in the ISABELA 1 trial and 781 patients in the ISABELA 2 trial, respectively.

*Learnings*. The small sample size and relatively short duration in the phase II trial may have contributed to the failure to reproduce positive results in phase III. Furthermore, a bi-directional drug-drug interaction between ziritaxestat and nintedanib that was only identified after the initiation of the ISABELA trials was likely an important contributor to the failure of the studies. Phase II trials should allow background antifibrotic therapy in order not to miss potential pharmacogenomic interactions.

*STARSCAPE*. Pentraxin-2, also known as serum amyloid P, is an endogenous blood protein that plays a key role in regulating wound healing and fibrosis via modulation of monocyte differentiation [11]. Pentraxin-2 knockout mice develop exaggerated pulmonary fibrosis following bleomycin-induced lung injury [12], and levels of Pentraxin-2 are reduced in the plasma of patients with IPF [13]. A phase II, randomized, double-blind, placebo-controlled trial showing that treatment with zinpentraxin alfa was associated with slower decline in FVC and stabilization of six-minute walk distance (6MWD) over 28 weeks [14] formed the rationale for the phase III STARSCAPE program [15]. Six hundred-sixty-four patients with IPF were randomized 1 : 1 to intravenous zinpetraxin alfa 10 mg/kg every 4 weeks or matching placebo. Background antifibrotic therapy was allowed, with 39% of patients receiving pirfenidone, 44% receiving nintedanib and 17% receiving neither. The primary endpoint was the absolute change in FVC (ml) from baseline to week 52. The trial was terminated early following a prespecified futility analysis that showed no benefit of zinpetraxin alfa over placebo. Similarly, no treatment effect was observed on several secondary endpoints, including 6MWD, patient-reported outcomes and time to clinically significant events such as disease progression and acute exacerbation. Posthoc analysis of the prior phase II data revealed that the apparent treatment effect was driven by three outliers, as indicated by the different treatment effect when using mean vs. median change in FVC from baseline. Specifically, two out of the three patients with the greatest annual decline in FVC (>2000 ml) were in the placebo arm, which greatly impacted the mean FVC decline in the placebo arm. After removal of the outliers from data analysis, the FVC slopes were similar in the zinpentraxin alfa and placebo arms, indicating no treatment effect.

*Learnings.* Careful data monitoring and quality control of spirometry during the trial; importance of analyzing data at patient level and routinely performing sensitivity analyses that exclude outliers; preferential use in FVC data analysis of median change from baseline (which is more robust to outliers than mean change) or mixed effect models that incorporate all data collected over the course of the study and which are less affected by individual outlying measurements.

*ZEPHYRUS-1 and ZEPHYRUS-2*. Pamrevlumab is a fully recombinant human monoclonal antibody against connective tissue growth factor (CTGF), a secreted matricellular protein that modulates several biological activities associated with tissue remodeling and fibrosis, including cell adhesion and migration, angiogenesis, myofibroblast activation, and extracellular matrix deposition [16]. In a murine model of radiation-induced pulmonary fibrosis, pamrevlumab has been shown to attenuate and even reverse lung remodeling [16]. In PRAISE, a phase II, randomized, double-blind, placebo-controlled trial, patients with IPF were randomly assigned (1 : 1) to intravenous pamrevlumab 30 mg/kg (*n* = 50) or matched placebo (*n* = 53) every three weeks over 48 weeks [17]. The primary endpoint was the change in the percentage of predicted FVC from baseline to week 48. Notably, antifibrotic therapy was not allowed. Pamrevlumab reduced the decline in the percentage of predicted FVC by 60% at week 48; the proportion of patients experiencing disease progression was also significantly lower in the pamrevlumab arm than in the placebo arm at week 48 (10% vs. 31%). The phase III clinical developmental program of pamrevlumab consisted of two studies, ZEPHYRUS-1 and ZEPHYRUS-2. In ZEPHYRUS-1, a double-blind, placebo-controlled trial, 356 patients with IPF were randomized 1 : 1 to either pamrevlumab or placebo for 48 weeks [18]. The study did not meet the primary endpoint of absolute change in FVC from baseline to week 48, as the mean FVC decline was 260 ml in the pamrevlumab arm compared to 330 ml in the placebo arm (*P* = 0.29). Similarly, there were no significant between-group differences in any of the secondary outcomes, including time to disease progression (FVC percentage predicted decline of ≥10% or death) and patient-reported outcomes was similarly not met. Based on the results of ZEPHYRUS-1, the decision to discontinue ZEPHYRUS-2 was made.

*Learnings*. Handling of missing data in small early phase trials can disproportionately influence estimates of effect size. Phase III studies should be powered using conservative estimates of effect size to ensure that smaller magnitudes of benefit are not overlooked in phase III trials. Excluding patients taking background antifibrotic therapy appreciably slows phase III trial recruitment.

## ADDRESSING CHALLENGES IN LATE-PHASE CLINICAL TRIALS IN IDIOPATHIC PULMONARY FIBROSIS

### Endpoint selection

Endpoint selection is critically important in the design, execution, and success of clinical trials; in addition, endpoints have the potential to influence regulatory approval and funding decisions and may improve patient care. In recent years, there has been vivid discussion on which endpoints reflect most reliably whether pharmacological interventions provide clinically meaningful benefit to patients with IPF.

Change in FVC over 1 year has been used as a primary endpoint in most RCTs in IPF based on its correlation with increased risk of mortality [19] and the shared view that studies with mortality as an endpoint are impracticable [20]. However, earlier identification of FVC decline would have the potential to accelerate early-phase studies. Khan and co-workers performed a systematic review and meta-analysis using individual patient data from 10 IPF clinical trials (*n* = 1819) to explore the relationship between short-term change in commonly measured physiological variables, namely FVC, DL_CO_, and 6-MWD, and clinically relevant outcomes [21^▪^]. They found that each 2.5% relative decline in FVC over 3 months increases the risk of mortality by 15% and the risk of disease progression by 30%. In addition, they found that an optimal threshold of 5.7% in FVC change at 3 months predicts mortality with an accuracy similar to that of a 10% FVC change in a 12-month period. Notably, the association between 3-month change in FVC and disease outcomes observed in the placebo arms were replicated in the treatment arms, supporting the prognostic significance of 3-month FVC change irrespective of antifibrotic therapy. However, using a three-month FVC decline as primary efficacy outcome would require approximately twice as many patients as traditional 12-month trials, and raises concerns on durability of the effect, as shown by the GLPG1690 (ziritaxestat) trials where the favorable effect on three-month FVC change observed in the phase II trial [8] was not confirmed in the 12-month phase III trial [10]. Three-month duration trials may also not be sufficiently long to reveal adverse events or unfavorable effects on survival.

The future of IPF trials was the topic of a symposium held in June 2023, which aimed at aligning scientific objectives and patient needs [22^▪▪^]. Indeed, while approved antifibrotic therapies slow down the rate of functional decline, they neither halt disease progression nor do they improve symptoms and quality of life. Patient reported outcomes (PRO), such as disease-related symptoms, provide direct assessment of how patients ‘feel’ by capturing their lived experiences directly from them. Similarly, assessments such as the 6-min walk test (6MWT) directly measure how patients ‘function’. The discussants also highlighted the possibility of replacing FVC as the primary endpoint by composite endpoints, which may aggregate into a single endpoint multiple outcomes, including change in lung function, exercise capacity (i.e., 6MWT), quality of life (i.e., dyspnea and cough), imaging and survival. Another such example is the combination of change in FVC of at least 10% and nonelective respiratory hospitalization, which have been shown to capture different domains of disease progression, and thus may increase statistical efficiency in time to event analyses due to the higher event rates and possibly smaller sample size [23]. While the FDA supported the implementation of composite endpoints in clinical trials of IPF, a number of complexities must be considered, including potential overlap of components, component equivalence based on their clinical meaningfulness and thus interpretation of the results [22^▪▪^].

### Innovative approaches to clinical trial design

*External controls*. External control arms (ECAs) are cohorts of control patients that derive from data sources external to a single-arm trial such as historical RCTs, registries or electronic health records (EHRs). They are increasingly used to assess treatment efficacy in clinical trials owing to their potential to reduce study duration and cost, increase generalizability of findings outside the setting of a clinical trial and mitigate the ethical challenges presented by (true) placebo arms [24]. Recently, Swaminathan *et al.* developed ECAs to mirror the BMS-986020 phase II RCT control arm [25^▪^] by applying different statistical approaches to patient data from previous RCTs in IPF, namely ACE-IPF [26], PANTHER-IPF [27] and STEP-IPF [28] as well as real-world data of IPF patients enrolled in the Pulmonary Fibrosis Foundation Patient Registry [29] and electronic medical records of IPF patients from the Duke University. The authors found that ECAs generated from historical RCTs closely matched the disease progression observed in the BMS-986020 placebo arm whereas controls originated from real-world datasets did not, suggesting caution in the use of real-world ECs to integrate future RCTs in IPF. However, the RCT data used by Swaminathan *et al.* predates the approval of pirfenidone and nintedanib to mirror more closely the BMS-986020 trial, which also excluded patients receiving antifibrotic therapy, whereas the majority of patients derived from real-world datasets were exposed to antifibrotic therapy due to the changes in treatment patterns that have occurred over time. The suitability of including ECs in RCT in IPF is supported by a recent phase II trial investigating the safety and efficacy of BI1015550 (nerandomilast), an oral preferential inhibitor of the PDE4B subtype in patients with IPF [30]. Further evaluations of ECs using more recent clinical trial data sources, including patients allowed background antifibrotic treatment as well as the exploration of endpoints beyond FVC, such as hospitalization, quality of life and death, are warranted.

*Adaptive multiinterventional trial platform*. In IPF, there is a long drug development pipeline, but there is also an urgent need to assess the effectiveness and safety of investigational drugs within a reasonably short time frame. Typical limitations of conventional RCTs include the need for large and prespecified sample sizes and the possibility to evaluate only one investigational drug at a time using a treatment and a control arm over a fixed study duration. In addition, by enrolling highly selected patients by virtue of strict inclusion and exclusion criteria, conventional RCTs produce data that may not be generalizable to real-word populations. The Randomized Embedded Multifactorial Adaptive Platform (REMAP) has the potential to maximize the efficiency of clinical trials by evaluating simultaneously multiple interventions (pharmacological or nonpharmacological), with patients randomized to multiple treatment domains and with a study protocol that is embedded in routine patient care, thus allowing a fast recruitment; moreover, the trial design may be changed and adapted as more information is gathered (i.e., removal of ineffective interventions) so that it is as effective and efficient as possible [31]. REMAP-CAP, a similar study investigating novel treatments in community acquired pneumonia [32], has been highly important in identifying effective (and ineffective) treatments for patients with Covid-19 pneumonia while CONQUEST (NCT06195072) is an adaptive trial platform for scleroderma associated interstitial lung disease that has recently started recruitment.

### Enrichment strategies

In clinical trials of IPF, disease progression is necessary in order to demonstrate treatment efficacy; therefore, the identification and enrolment of patients more likely to progress is instrumental to the design and conductance of a successful clinical trial. The application of machine learning and artificial intelligence analysis to the assessment of computerized tomography images from individuals with fibrotic lung disease promises to improve identification of usual interstitial pneumonia (UIP)/IPF and to enable enrichment of studies with more rapidly progressive disease. Humphries and colleagues [33^▪▪^] developed a multiple instance learning algorithm that reliably identifies individuals with UIP, an increased mortality risk and more rapid FVC decline over 52 weeks. Muhunthan *et al.* used a different deep learning-based segmentation method applied to the prospective PROFILE cohort to successfully identify individuals at increased 1-year risk of death or disease progression even when corrected for baseline disease severity [34^▪^]. The increased availability of high-throughput unbiased proteomic analysis is enabling the development of serum-based multiprotein signatures that identify individuals with IPF at risk of disease progression [35^▪^]. Meanwhile, the NIH-funded PRECISIONS (NCT04300920) study is building on genetic-based insights to enroll IPF patients with a specific polymorphism in the TOLLIP gene to be tested in a randomized controlled trial of the antioxidant N-acetyl cysteine [36]. Thus, the application of radiomic, genetic and proteomics is likely to be increasingly used to enable effective enrichment of IPF trials in the future.

## CONCLUSION

Several promising drugs are currently in development in IPF mirroring the urgent need for better treatments for patients living with this terrible disease. A number of innovative approaches have the potential to increase the likelihood of success of late-phase clinical trials of IPF, including novel trial designs, alternative primary outcomes and more efficient enrolment of study participants. However, a better understanding of the mechanisms driving lung fibrosis and an accelerated process of drug development remain instrumental to the identification of truly efficacious therapies for people living with IPF.

## Acknowledgements

*None.*

### Financial support and sponsorship

*None*

### Conflicts of interest

*P.S., via his institution, has received funding from Boehringer Ingelheim, PPM Services and Roche; and consultancy or speaker fees from Astra Zeneca, Boehringer Ingelheim, BMS, Chiesi, CSL Behring, Galapagos, GlycoCore Pharma, JucaBio, Menarini, Merck, Pieris, PPM Services, Structure Therapeutics and Trevi.*

*T.M.M., via his institution, has received industry-academic funding from Astra Zeneca and GlaxoSmithKline R&D; and consultancy or speaker fees from Astra Zeneca, Bayer, Boehringer Ingelheim, BMS, CSL Behring, Endeavor, Fibrogen, Galapagos, Galecto, GlaxoSmithKline, IQVIA, Merck, Pliant, Pfizer, Qureight, Roche, Sanofi-Aventis, Structure Therapeutics, Trevi and Vicore. T.M.M. is supported by a British Lung Foundation Chair in Respiratory Research (C17-3).*

## REFERENCES AND RECOMMENDED READING

Papers of particular interest, published within the annual period of review, have been highlighted as:

▪ of special interest
▪▪ of outstanding interest
